# Severe Bilateral Optic Neuritis: A Rare Presentation of Clinically Isolated Syndrome

**DOI:** 10.7759/cureus.11135

**Published:** 2020-10-24

**Authors:** Akash Khetpal, Ranjeet Kumar, Neelam Khetpal

**Affiliations:** 1 Internal Medicine, Dow University of Health Sciences, Karachi, PAK; 2 Internal Medicine, AdventHealth Florida Hospital, Orlando, USA

**Keywords:** multiple sclerosis, bilateral optic neuritis, clinically isolated syndrome

## Abstract

Multiple sclerosis (MS) is a disabling disease involving the myelin sheath of neurons in the brain and spinal cord. Optic neuritis (ON) is one of the common presentations of MS, and it typically presents as acute unilateral ON. In the absence of a prior history of MS, the first episode of clinically determined acute ON is considered as a clinically isolated syndrome (CIS). CIS presenting with bilateral acute ON resulting in complete vision loss is very rare. We present a case of a young female patient who presented with bilateral progressive vision loss and was ultimately found to have ON in the absence of a prior history of MS. Important differentials were ruled out. Significant improvement was observed in the patient with high doses of intravenous methylprednisolone. As compared to oral prednisone, intravenous steroids have been shown to be more effective in the treatment of ON in the Optic Neuritis Treatment Trial.

## Introduction

Multiple sclerosis (MS) is a demyelinating disease commonly affecting oligodendrocytes of nerves in the brain and spinal cord. Oligodendrocytes produce and maintain myelin sheaths in the central nervous system. The characteristic features of MS are immune-mediated inflammation and loss of protective myelin sheath, which is essential for the rapid conduction of nerve signals.

The presentation of MS is diverse; however, certain symptoms are commonly present in all affected patients [1]. Common clinical manifestations of MS are sensory-motor deficits, visual symptoms, and weakness and fatigue. Early ocular signs of MS can be optic neuritis (ON), blurred vision, or diplopia [2].

ON is the initial presentation in 15-20% of cases of MS, and it develops at some point during the course of disease in 38-50% of the patients [3]. ON is almost always acute and unilateral in adults; simultaneous bilateral ON is an exceedingly rare presentation, especially in those patients with no prior history of MS.

## Case presentation

A 25-year-old obese female with a prior medical history of sickle cell trait presented with an initial complaint of headache for one month. The patient had never been admitted before for any sickle cell-related complications. She had localized headache to the bilateral frontal regions and described it as a constant, moderate to severe, pressure-like, and non-radiating pain. The pain worsened with ocular movement and relieved minimally with over-the-counter pain-killers. Her headache was not associated with fever, nausea, vomiting, photophobia, lacrimation, weakness, numbness, gait issues, memory problems, or any recent upper respiratory tract infection. She was the mother of one child and was not on oral contraceptive pills (OCPs). No history of acute vision loss was present in the family.

**Figure 1:**
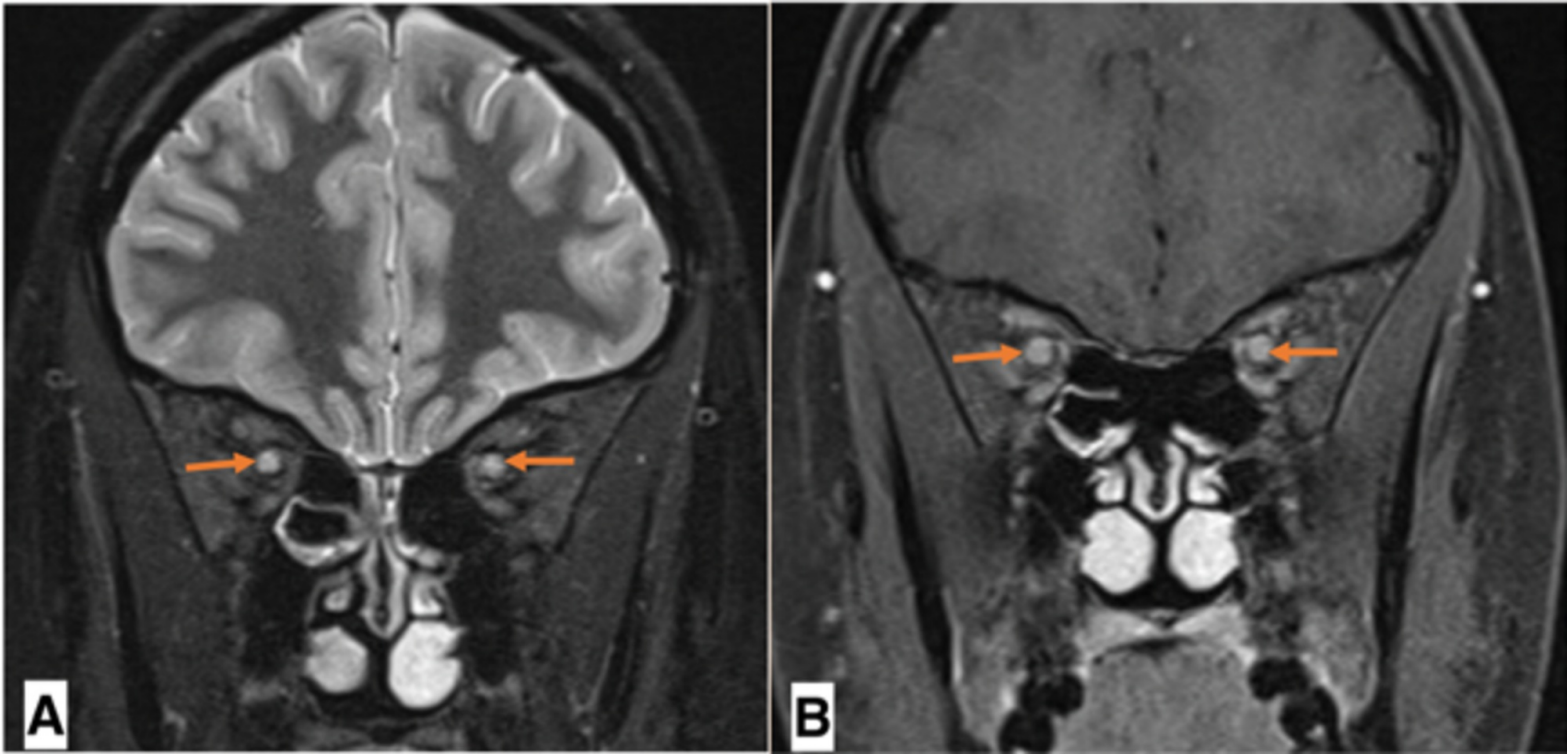
MRI of the orbit A: bilateral 'orbital' optic nerve segment (arrows) T2 prolongation and pathological enhancement. B: T2 prolongation and pathological enhancement of ‘prechiasmatic' segment of optic nerve (arrows) MRI: magnetic resonance imaging

On physical exam, she had painful ocular movements and limited visual acuity to only a slight perception of bright light. The relative afferent pupillary defect (RAPD) could not be assessed due to the involvement of both eyes. Later, she developed a progressive decrease in visual acuity that culminated in bilateral complete vision loss. Various investigations were done to reach an accurate diagnosis and rule out other possible diagnoses, most importantly neuromyelitis optica (NMO) and idiopathic intracranial hypertension (IIH). MRI of the brain and spinal cord did not reveal any pathological neuronal processes. On fundoscopic examination, she was found to have severe bilateral papilledema. Lumbar puncture revealed normal-to-slightly-raised opening pressure and aseptic meningitis-like cell count with differentials. MRI of the orbit, later on, confirmed the diagnosis of bilateral ON (Figure 1). The available cerebrospinal fluid (CSF) specimen was then tested for oligoclonal bands, which also came back positive. Diagnosis of a clinically isolated syndrome (CIS) was confirmed in light of the symptoms and MRI and CSF findings.

Following the guidelines of Optic Neuritis Treatment Trial (ONTT), a landmark study for managing the acute onset of ON, we gave our patient high doses of intravenous methylprednisolone during her hospitalization. Significant improvement was noticed within the first few days of treatment initiation as determined by her ability to appreciate colors, shapes, and sources of light.

## Discussion

MS is a debilitating disease characterized by a relapsing and remitting course in the majority of patients. Visual disturbance is the most common initial presentation of MS [1]. The initial visual abnormalities associated with MS are acute and unilateral ON, blurred vision, diplopia, chronic bilateral uveitis, facial palsy, paresthesia, or numbness in one or more areas supplied by the trigeminal nerve [2].

The incidence of ON is approximately 115 cases per 100,000 people. Patients usually present in their third or fourth decade of life, and the mean age of presentation is 32 years. ON is more common in women than men. The vision loss is associated with pain on the ocular movement, which is thought to be due to muscle traction on the inflamed optic nerve at the annulus of Zinn [3]. ON is usually inflammatory, but it can occur following an infection caused by viruses (influenza, mumps, and measles), bacteria (*Bartonella henselae, *tuberculosis, syphilis*, *and* Borrelia burgdorferi*), fungi (*cryptococci, candida, Histoplasma, aspergillus, *and* *mucormycosis) and parasites (*Toxoplasma gondii, Toxocara canis, Onchocerca volvulus, *malaria, and *Echinococcus*). In mumps, ocular involvement typically presents as follicular conjunctivitis, episcleritis, dacryoadenitis, keratitis, scleritis, anterior uveitis, choroiditis, or paralysis of extraocular muscles (abducens nerve palsy); however, in rare cases, mumps can also present with bilateral ON [4]. In July 2000, the International Panel on the Diagnosis of MS issued a set of criteria for making a diagnosis of MS [5]. Based on the above-mentioned criteria, Table 1 lays out the clinical presentation related to the diagnosis of MS (left column) as well as the additional data needed to make an MS diagnosis (right column).

Treatment of ON with steroids was evaluated in the ONTT, where patients were randomly assigned to either oral prednisone or intravenous methylprednisolone groups followed by oral prednisone or oral placebo [6]. Accelerated recovery of visual function was found with intravenous methylprednisolone. Intravenous methylprednisolone was also found to reduce the risk of conversion to MS within the first two years as compared to either placebo or oral prednisone. In ONTT, the oral prednisone was found to have a higher two-year risk of recurrent ON, when compared with intravenous steroid therapy or placebo. Also, at 10 years, the risk of recurrent ON remained higher in the oral prednisone group when compared with the intravenous-treated group [6]. Patients poorly responding to steroids can be treated with intravenous immunoglobulins or plasma exchange [7,8].

**Table 1:** Diagnostic criteria of MS MS: multiple sclerosis; MRI: magnetic resonance imaging; CSF: cerebrospinal fluid; VEP: visual evoked potential

Clinical presentation	Additional data needed for MS diagnosis
Objective clinical evidence of 2 or more lesions	
Two or more attacks; objective clinical evidence of 1 lesion	Dissemination in space, demonstrated by MRI or two or more MRI‐detected lesions consistent with MS plus positive CSF, or await further clinical attack implicating a different site
One attack; objective clinical evidence of 2 or more lesions	Dissemination in time, demonstrated by MRI or a second clinical attack
One attack; objective clinical evidence of 1 lesion (monosymptomatic presentation; clinically isolated syndrome)	Dissemination in space, demonstrated by MRI or two or more MRI‐detected lesions consistent with MS plus positive CSF, and dissemination in time, demonstrated by MRI or a second clinical attack
Insidious neurological progression suggestive of MS	Positive CSF and dissemination in space, demonstrated by 1) nine or more T2 lesions in the brain, 2) 2 or more lesions in the spinal cord, or 3) 4–8 brain plus 1 spinal cord lesion or abnormal VEP associated with 4–8 brain lesions, or with fewer than 4 brain lesions plus 1 spinal cord lesion demonstrated by MRI, and dissemination in time, demonstrated by MRI or continued progression for 1 year

In the absence of a prior history of MS, the first episode of clinically or radiologically determined acute ON is considered as a clinically or radiologically isolated syndrome, respectively. It is important for the patient to know about the likelihood of MS developing after one episode and its prognosis [9]. CIS presenting with ON generally has a better prognosis and, as determined by Jacob et al., patients with CIS have a 28% chance of developing clinically definite MS (CDMS) in the next five to six years [10].

In most cases, CIS is mild and may resolve without therapeutic intervention. Features that necessitates treatment are severe visual loss, pain in ON, marked motor dysfunction, ataxia, or vertigo in the spinal cord and brainstem syndromes. High-dose intravenous methylprednisolone shortens the duration of the visual deficit but not visual outcome after one year [11].

## Conclusions

Although ON in MS is not uncommon, the occurrence of bilateral acute ON without any prior history of MS is an unusual presentation. Intravenous methylprednisone is still considered the standard treatment for acute-onset MS and CIS. Intravenous methylprednisone has been shown to decrease the duration of visual loss associated with CIS. However, more data is needed regarding the potential use of disease-modifying therapies used in the treatment of CDMS.

## References

[1] Poser S, Wikström J, Bauer HJ (1979). Clinical data and the identification of special forms of multiple sclerosis in 1271 cases studied with a standardized documentation system. J Neurol Sci.

[2] Roodhooft JM (2009). Ocular problems in early stages of multiple sclerosis. Bull Soc Belge Ophtalmol.

[3] Gilbert ME, Sergott RC (2007). New directions in optic neuritis and multiple sclerosis. Curr Neurol Neurosci Rep.

[4] Khan B, Nasir S, Hanif S (2020). Bilateral optic neuritis: a rare complication of mumps. Cureus.

[5] McDonald WI, Compston A, Edan G (2001). Recommended diagnostic criteria for multiple sclerosis: guidelines from the International Panel on the diagnosis of multiple sclerosis. Ann Neurol.

[6] Beck RW, Cleary PA, Anderson MM Jr (1992). A randomized, controlled trial of corticosteroids in the treatment of acute optic neuritis. The Optic Neuritis Study Group. N Engl J Med.

[7] Roed HG, Langkilde A, Sellebjerg F, Lauritzen M, Bang P, Mørup A, Frederiksen JL (2005). A double-blind, randomized trial of IV immunoglobulin treatment in acute optic neuritis. Neurology.

[8] Ruprecht K, Klinker E, Dintelmann T, Rieckmann P, Gold R (2004). Plasma exchange for severe optic neuritis: treatment of 10 patients. Neurology.

[9] Tintoré M, Rovira A, Rio J (2005). Is optic neuritis more benign than other first attacks in multiple sclerosis?. Ann Neurol.

[10] Jacobs LD, Kaba SE, Miller CM, Priore RL, Brownscheidle CM (1997). Correlation of clinical, magnetic resonance imaging, and cerebrospinal fluid findings in optic neuritis. Ann Neurol.

[11] Miller DH, Chard DT, Ciccarelli O (2012). Clinically isolated syndromes. Lancet Neurol.

